# Using single cell cultivation system for on-chip monitoring of the interdivision timer in *Chlamydomonas reinhardtii *cell cycle

**DOI:** 10.1186/1477-3155-8-23

**Published:** 2010-09-25

**Authors:** Kazunori Matsumura, Toshiki Yagi, Akihiro Hattori, Mikhail Soloviev, Kenji Yasuda

**Affiliations:** 1 Department of Life Sciences, Graduate School of Arts and Sciences, University of Tokyo, 3-8-1 Komaba, Meguro, Tokyo 153-8902, Japan; 2 Structural Biology, Graduate School of Science, Kyoto University, Oiwake, Kitashirakawa, Sakyo-ku, Kyoto 606-8502, Japan; 3 Kanagawa Academy of Science and Technology, KSP East 310, 3-2-1 Sakado, Takatsu-ku, Kawasaki, Kanagawa 213-0012, Japan; 4 School of Biological Sciences, Royal Holloway University of London, Egham, Surrey TW20 0EX, UK; 5 Division of Biosystems, Institute of Biomaterials and Bioengineering, Tokyo Medical and Dental University, Tokyo; 6 2-3-10 Kanda-Surugadai, Chiyoda, Tokyo 101-0062, Japan

## Abstract

Regulation of cell cycle progression in changing environments is vital for cell survival and maintenance, and different regulation mechanisms based on cell size and cell cycle time have been proposed. To determine the mechanism of cell cycle regulation in the unicellular green algae *Chlamydomonas reinhardtii*, we developed an on-chip single-cell cultivation system that allows for the strict control of the extracellular environment. We divided the *Chlamydomonas *cell cycle into interdivision and division phases on the basis of changes in cell size and found that, regardless of the amount of photosynthetically active radiation (PAR) and the extent of illumination, the length of the interdivision phase was inversely proportional to the rate of increase of cell volume. Their product remains constant indicating the existence of an 'interdivision timer'. The length of the division phase, in contrast, remained nearly constant. Cells cultivated under light-dark-light conditions did not divide unless they had grown to twice their initial volume during the first light period. This indicates the existence of a 'commitment sizer'. The ratio of the cell volume at the beginning of the division phase to the initial cell volume determined the number of daughter cells, indicating the existence of a 'mitotic sizer'.

## Background

Proliferating eukaryotic cells maintain a relatively constant size by coordinating their growth with the progression of the cell cycle [1], and their responses to changing environmental conditions, which are mainly evident in the G_1 _phase [2-4]. When sufficient nutrients are not available, cells delay their progress through the G_1 _phase or enter a specialized resting state known as G_0 _[5]. If sufficient nutrients are available, cells in early G_1 _or G_0 _phase pass through a control point that in the yeast cell cycle is referred to as the 'start' [6,7] and in the mammalian cell cycle is referred to as the 'restriction point' [5,8]. After passing through this control point, cells are committed to initiating DNA replication and proceed to the S phase even if sufficient nutrients are no longer available [5,9]. Both size-dependent and time-dependent controllers have been proposed to determine the length of the G_1 _phase [7]: the 'sizer' determines whether the cell has reached the threshold size needed to progress to the next phase, and the 'timer' determines whether the cells have been in the G_1 _phase long enough. The exact molecular mechanisms behind these controllers remain unknown because experimentalists have not been able to control environmental conditions, such as nutrient conditions, cell-cell interactions and cell cycle phase synchronization, well enough for their effects to be analyzed quantitatively.

**Figure 1:**
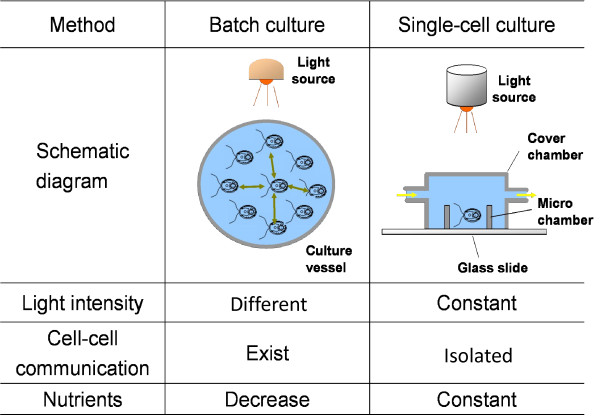
**Comparison between batch culture and single-cell culture methods**.

Several groups used microfluidic-type devices for studying the mechanisms of cell cycle regulation and division control under the controlled conditions [10-18]. We have earlier developed an on-chip cultivation system for use with the unicellular green algae *Chlamydomonas reinhardtii*. The photosynthetic algae *Chlamydomonas *uses light as the source of energy. This property allows one to easily manipulate and vary the amount of energy supplied to the cells by varying the light, whilst maintaining other environmental conditions (such as the carbon dioxide concentration in the medium) unchanged [19,20]. Our on-chip system prevents indirect cell-cell communication (i.e. via chemical secretion) by continuously perfusing individual microchambers containing single cells with fresh medium (Figure 1).

The *Chlamydomonas *cell cycle has a long G_1 _phase during which cells can grow to more than twice their initial size [21,22]. *Chlamydomonas *divides by multiple fission [23]. The long G_1 _phase is followed by a short division phase in which mother cells alternate rapidly between S and M phases [23]. The G_1 _phase was found to have two regulatory points coordinating the progression of the cell cycle with cell growth [24]. One is the 'primary arrest point' at the beginning of the phase, at which the cell cycle becomes blocked if the cells cultured in minimal medium are devoid of light, and is conceptually similar to the 'start' in the yeast cell cycle or the 'restriction point' in the mammalian cell cycle. The other is the 'transition point' late in the phase, at which cells are committed to completing the division cycle regardless of subsequent illumination. Previous studies have suggested that a 'timer' and/or 'sizer' are involved in *Chlamydomonas *cell cycle regulation [21-23,25]. Although the cell cycle regulatory genes have been characterized [26,27], no research has been conducted to investigate the coordination of cell growth and cell cycle progression in *Chlamydomonas *under a fully controlled environment at single cell level.

In this study we used our on-chip cultivation system to examine the duration of cell cycle phases and to measure the cell volume of individual *Chlamydomonas *cells under different nutrient conditions produced by defined illumination (time and intensity of light exposure). We found that the length of the interdivision phase (comprising the G_1_, G _2_, and S phases) was inversely proportional to the rate at which cell volume increased during the light period. We also found that the passage of the cells through the primary arrest point was dependent on whether they had attained twice their initial cell volume by the end of the light exposure period. Our results also indicate that a mitotic 'sizer' determines the number of daughter cells by monitoring the cell growth during the interdivision phase.

## Materials and methods

### Strain and culture conditions

We used a central-pair-lacking (non-motile mutant) strain of *Chlamydomonas reinhardtii*, the *pf18^+ ^*strain. We used a minimal medium throughout our experiments in order to exclude energy intake other than the exposure to light. This was based on SG medium [28], except that MnSO_4_•5H_2_O was substituted for MnSO_4_•4H_2_O. A stock solution was prepared by adding K_2_HPO_4 _(0.1 g), KH_2_PO_4 _(0.1 g), NH_4_NO_3 _(0.3 g), MgSO_4_•7H_2_O (0.3 g), CaCl_2 _(0.04 g), FeCl_3_•6H_2_O (0.01 g), sodium citrate-2H_2_O (0.5 g), and 10 ml of a trace metal solution containing H_3_BO_3 _(0.1 g/L), ZnSO_4_•7H_2_O (0.1 g/L), MnSO_4_•5H_2_O (0.43 g/L), CoCl_2_-6H_2_O (0.02 g/L), Na_2_MnO_4_•2H_2_O (0.02 g/L), and CuSO_4_•5H_2_O (0.04 g/L) to 1 L of distilled water. All chemicals were from Wako Pure Chemical Industries, Ltd. (Osaka, Japan). Cells obtained from agar slants were put into 10 ml of minimal medium in a 15-ml test tube and incubated at room temperature (25°C) with aeration by filtered fresh air and exposure to continuous light. The cells used for on-chip cultivation were taken from the culture in the early-log phase (≈10^4 ^cells/ml).

### On-chip single-cell cultivation system

**Figure 2:**
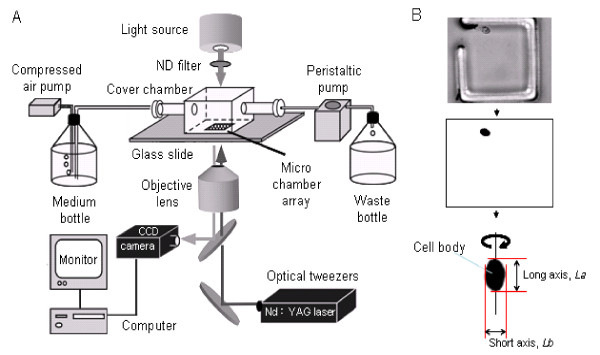
**A: Schematic diagram of the on-chip single-cell cultivation system for *Chlamydomonas***. **B**: Image analysis protocol. (*top*) Micrographs are acquired using the time-lapse recording. (*middle*) The micrograph is digitized and cell contours are determined by applying a threshold filter. (*bottom*) Cell volume is calculated from the cross-sectional area assuming axisymmetrical shape of *Chlamydomonas *cells.

The on-chip single-cell cultivation system that we used (Figure 2A) was the same as that described previously [19,20]. The system is based around a single-cell cultivation unit consisting of a microcultivation chamber array made of a 30-μm thick photoresist on a 0.2-mm thick glass slide. The latter is attached to a cover chamber through which the minimal medium is supplied and circulated. Cells are recorded with a time-lapse recording unit with a bright-field optical microscopy system (IX-70 inverted microscope with oil-immersion objective lens, 100×, NA = 1.35, Olympus, Tokyo) equipped with a CCD camera (CS230, Olympus, Tokyo). An optical tweezers unit (1064-nm Nd:YAG laser, T20-8 S, Spectra-physics, Mountain View, CA) was used to manipulate individual *Chlamydomonas *cells.

The microchamber array was made of a negative photoresist (SU-8 25, Microlithography Chemical, Newton, MA) and was microfabricated using photolithography on a glass slide (the exposed part remained on the glass). Each microchamber in the array was a square area surrounded by 60 μm long and 30 μm high walls, one of which had a 20 μm wide gate [19,20].

By enclosing the cells in microchambers we were able to observe them in a liquid medium for a long time without the cells escaping the field of vision of the microscope. Daughter cells produced by cell division were removed from the chamber through the gate. The halogen light source for microscopy illumination was used to illuminate cells and therefore provided the energy source for the cells. The amount of photosynthetically active radiation (PAR) used for cultivation ranged from 10 to 200 μmol m^-2 ^s^-1 ^(photosynthetic photon flux density) and was adjusted by using a combination of ND filters (45-ND6, Olympus, Tokyo; and XB119/32R, Omega Optical, Brattleboro, VT) in order to maintain the shape of the spectrum of the light source. The illumination intensity on the microscope stage was measured with a luminometer (LM-332, AS ONE, Osaka). The radiant flux of the 1064-nm laser for the optical tweezers was less than 11 mW, which is the highest flux that did not cause any damage to the cells (data not shown).

### Microcultivation procedure

Ten microlitres of a 1 mg/ml BSA solution was applied to the microchamber array plate to prevent cells from clinging to the microchamber surface. After 30 min of incubation, 10 μl of *Chlamydomonas *culture was transferred onto the microchamber array plate. Following this a cover chamber was placed on the microchamber array plate and was sealed with polydimethylsiloxane (Dow Corning, Midland, MI). The chamber was connected to a reservoir containing the minimal medium. In order to prevent contamination all procedures were done on a clean bench, and all the materials were autoclaved before cultivation commenced. The microchamber array chip with *Chlamydomonas *culture and the cover chamber was then positioned on the microscope stage and perfused with the minimal medium at 1 ml/min flow rate. We used second-generation samples for observations because the growth rate for the first generation was not stable. Following the division of the first-generation cell, one of the daughter cells was kept in each microchamber and the other cells were removed with optical tweezers. During the experiment we monitored the cell cycle and measured the number of divisions and the volume of each cell using time-lapse recording.

### Image analysis

Cell volume was estimated based on the measured cell contours, as illustrated in Figure 2B. The cross-sectional area of the cells was first calculated from the 640 × 480-pixel images recorded at 30-s intervals. Each micrograph was digitized using an adequate threshold for recognition of cell contours using image analysis software (Scion Image, Scion Corp., Frederick, ML). Because *Chlamydomonas *cells are axially symmetrical, cell volume was then calculated by using spreadsheet software to rotate the cross-sectional area around the longer axis (*L_a_) *in the plane of the cross section, i.e., the estimated cell volume is equal to

(1) $$V=(4/3)\pi (L_{a}+L_{b})$$

where *L_b _*is the length of shorter axis of ellipsoidal body.

## Results

### Cell cycle phases under continuous illumination

**Figure 3:**
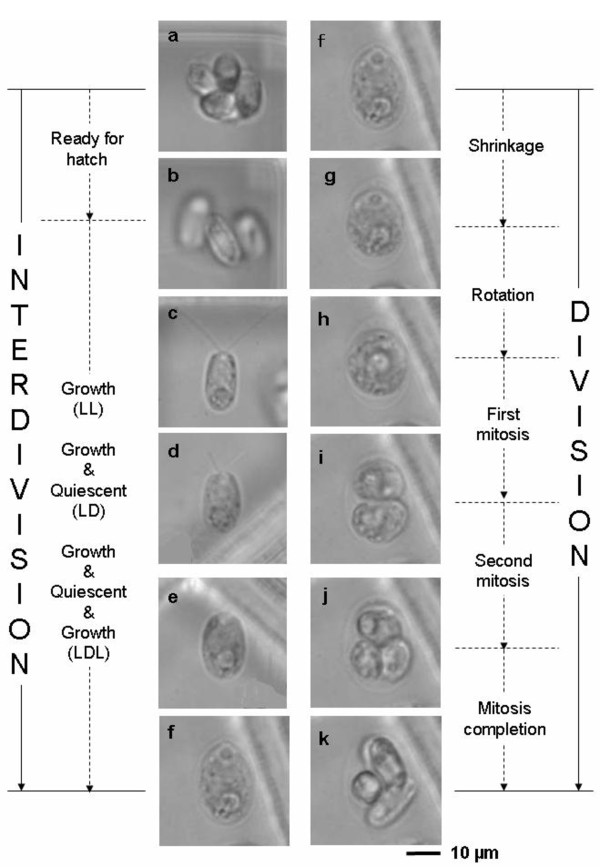
***Chlamydomonas reinhardtii *cell cycle**. Bright-field optical micrographs of cells. Cell cycle was divided into two phases: interdivision (left panels **a, b, c, d, e **and **f**) and division (right panels **f, g, h, i, j **and **k**). Interdivision phase consists of two subphases: ready-for-hatch (left panels **a **and **b**) and growth-after-hatching (left panels **c, d, e **and **f**). Division phase consists of five subphases: shrinkage (right panels **f **and **g**), rotation (right panels **g **and **h**), first mitosis (right panels **h **and **i**), second mitosis (right panels **i **and **j**), and mitosis-completion (right panels **j **and **k**). Images were acquired after long-term cultivation of cells under continuous light illumination (200 μmol m^-2 ^s^-1^).

The advantage of a single-cell cultivation method is that it enables to conveniently study and record changes in the size of individual cells as well as the duration of their cell cycle phases without the need for synchronous cell cultivation. Bright-field optical microscopy with a ×100 objective lens reveals the shapes of the cells with a spatial resolution of 0.2 μm, which is sufficient for identifying cell cycle phases, see Figure 2. We divided the *Chlamydomonas *cell cycle into two phases determined by changes in their outer shape: interdivision and division phases (Figure 3).

The interdivision phase consisted of two subphases called the 'ready-for-hatch' subphase and the 'growth-after-hatching' subphase (Figure 3). In the first subphase, the measured volumes of each of the daughter cells immediately after hatching appeared to be more than a quarter of their mother's final (maximum) cell volume (just before the mother cell entered the division phase). Therefore, daughter cells seem to have started their growth immediately following the mitosis of their mother cell. For example, when the final volume of a mother cell was 277 μm^3 ^(a quarter of which is 69 μm^3^), the measured volume of each hatched daughter was 87 μm^3^. Being unable to measure daughter cell volumes immediately following the mitosis, we used the value equal to one quarter of the mother cell maximum volume as the daughter's initial cell volume.

The division phase consisted of five subphases called the 'shrinkage', 'rotation', 'first-mitosis', 'second-mitosis', and 'mitosis-completion' (Figure 3). After cell growth ceased, cells detached their cell membrane from their cell wall and shrunk to form a spherical shape (the shrinkage subphase) [18]. They then rotated within their cell wall (the rotation subphase). After the mother cell divided twice, the shape of the four daughter cells changed from spherical to rod-like (the mitosis-completion subphase).

### Effect of illumination intensity on cell cycle phase duration

**Figure 4:**
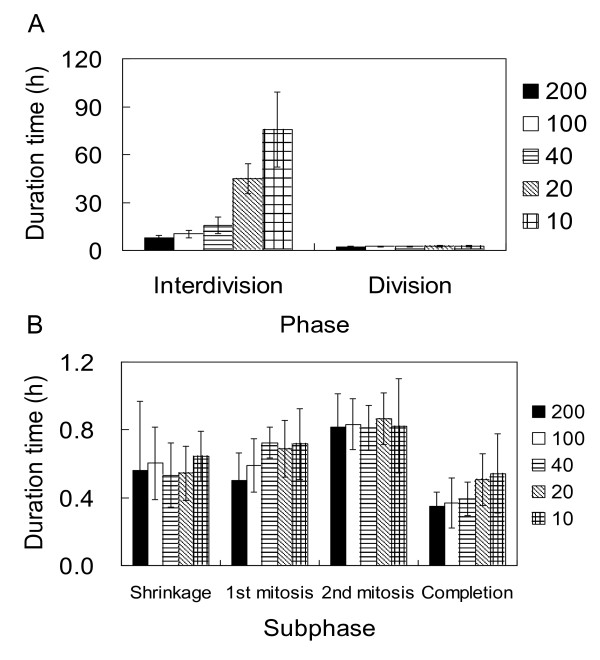
**Effect of the amount of photosynthetically active radiation (PAR) on the duration of the cell cycle phases and subphases**. Light intensities used for continuous illumination were 200 (filled bars), 100 (open bars), 40 (horizontally striped bars), 20 (hatched bars) and 10 (gridded bars) μmol m^-2 ^s^-1^. **A**: Duration of the interdivision and division phases. **B**: Duration of the division subphases.

We examined the effect of phothsynthetically active radiation (PAR) on the duration of each phase at a single-cell level (Figure 3). The amounts of (PAR) used for continuous illumination were 200 (N = 26), 100 (N = 26), 40 (N = 9), 20 (N = 8), and 10 (N = 10) μmol m^-2 ^s^-1^. Duration of the interdivision phase was inversely related to PAR, increasing by a factor of 8 as PAR decreased by a factor of 20 (Figure 4A). The standard deviation (SD) also differed depending on the light intensities, but the coefficients of variation (CV, a normalized measure of dispersion) were similar at each intensity setting (27%, 23%, 38%, 21%, and 31% at 200, 100, 40, 20, and 10 μmol m^-2 ^s^-1 ^respectively).

In contrast to the interdivision phase, duration of the subphases in the division phase did not change significantly with PAR (Figure 4B) or were independent on the cell volume (data not shown). We conclude therefore that PAR affects duration of the interdivision phase but not that of the following division phase.

### Effect of illumination intensity on cell growth during the interdivision phase

**Figure 5:**
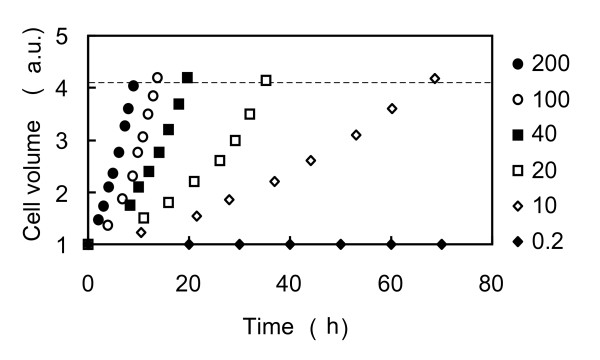
**Effect of PAR on cell growth during the interdivision phase**. Light intensities are as indicated (PAR values are in m^-2 ^s^-1^), these were the same as in Figure 4. White arrowheads indicate the beginning of the division phase. The dashed line shows the critical cell size for entering the division phase relative to the initial cell volume (4.0 ± 1.0, ± SD).

We examined the time course of changes in cell volume during the interdivision phase under different conditions of uninterrupted continuous light exposure. We found that the rate of increase in cell volume increases with PAR (Figure 5). Cells cultured at all PARs greater than 0.2 μmol m^-2 ^s^-1 ^entered the division phase when they grew to ~ 4.1 times their initial volume (white arrowheads in Figure 5). Although the cells cultured under 0.2 μmol m^-2 ^s^-1 ^neither grew nor divided, changes in PAR between 10 and 200 μmol m^-2 ^s^-1 ^affected the rate at which cell volume increased but did not affect the ratio of the final cell volume to the initial cell volume.

### Exponential growth model of cell volume during the interdivision phase

**Figure 6:**
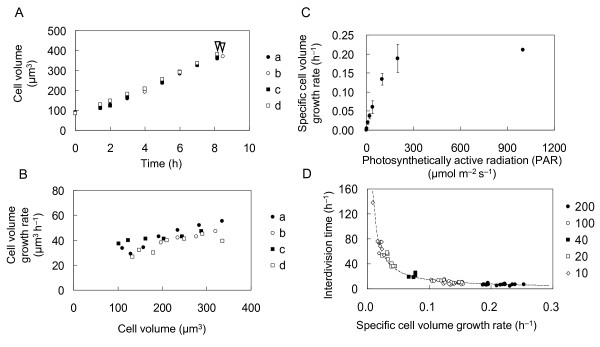
**Nonlinear increase of *Chlamydomonas *cell volume**. **A**: Time course of the daughter cell's volume increase under continuous illumination at 200 μmol m^-2 ^s^-1 ^(data for four cells, shown as "a", "b", "c" and "d"). White arrowheads indicate the beginning of the division phase. **B**: Volume increase rate as a function of cell volume calculated from data in panel A. **C**: Volume increase rate versus PAR. **D**: Relationship between the volume increase rate and duration of the interdivision phase for the cells cultured under continuous illumination with light intensities ranging from 10 to 200 μmol m^-2 ^s^-1^. The product of the volume increase rate and the interdivision phase duration equals 1.4. Light intensities are as indicated (PAR values are in m^-2 ^s^-1^).

In order to quantify the rate of cell volume increase, we cultured daughter cells in a microchamber under continuous illumination at 200 μmol m-2 s-1 (Figures 6A and 6B). The volume *V(t) *of individual *Chlamydomonas *cells increased exponentially:

(2) $$V(t)=V(0)e^{\mu t}$$

where *V(0) *is the initial cell volume, just after the completion of mitosis. The rates of cell volume increase (*μ*) were calculated for various light intensities:

(3) $$\mu =\frac{1}{T}\ln\left(\frac{V(T)}{V(0)}\right),$$

where *T *is the time from the completion of mitosis to the beginning of the division phase (interdivision phase duration) and *V(T) *is the cell volume just before entering the division phase (final cell volume), see Figure 6C. The rates of cell volume increase with PAR and reach a plateau when PAR reaches approximately 300 μmol m^-2 ^s^-1^. Duration of the interdivision phase (*T*) and the specific cell volume growth rate (*μ*) were inversely proportional (Figure 6D);

(4) μ⋅T=1.4

Combining equations (4) and (2) yields:

(5) V(T)/V(0)=4.1

This ratio determines a threshold value for cell size and is identical to the values measured experimentally (see Figure 5).

#### Cell cultivation under various continuous light (LL, Light-Light) conditions

**Figure 7:**
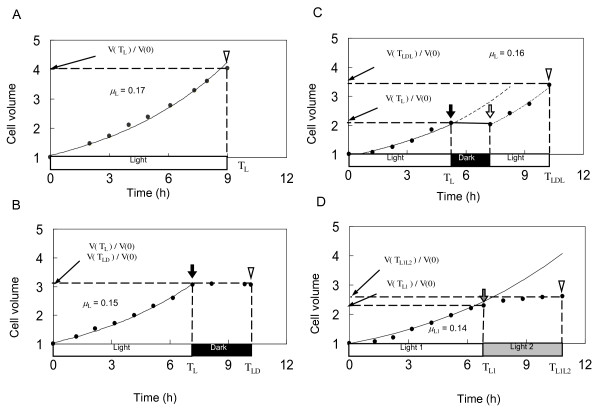
**Single-cell cultivation under various lighting conditions**. **A**: LL (continuous light) condition. PAR was 200 μmol m^-2 ^s^-1^. Cell volume is normalized to the initial cell volume. The white arrowhead indicates the time at which the cell enters the division phase. The solid line indicates an exponential fit; *μ_L _*is the volume increase rate given by *μ_L _*= (1/*T_L_*)ln(*V(T_L_)/V(0)*). **B**: LD condition. PAR was the same as in panel A. The light exposure was stopped at the time indicated by the black arrow; *T_L _*is the duration of the light exposure period. The cells entered the division phase at the time indicated by the white arrowhead. *T_LD _*is the interdivision phase duration under the LD condition, and *μ_L _*is the volume increase rate during the light period calculated as in panel A. **C**: LDL condition. PAR was the same as in panels A and B. The light exposure was stopped (black arrow) and restarted (white arrow) before the target single cell entered the division phase. The cell entered the division phase at the time indicated by the white arrowhead, before reaching 4.1 times its initial volume. *T_L _*is the first light period duration, *T_LDL _*is the interdivision phase duration under the LDL condition, and *μ_L _*is the volume increase rate during the light period. **D**: L1L2 illumination condition (i.e., first 200 μmol m^-2 ^s^-1 ^and then 100 μmol m^-2 ^s^-1^). The cell entered the division phase at the time indicated by the white arrowhead before reaching 4.1 times its initial volume. *T_L1 _*is the duration of the first light exposure period (L1), *T_L1L2 _*is the interdivision phase duration under the L1L2 condition, and *μ_L1 _*is the rate of cell volume increase during L1 period.

**Figure 8:**
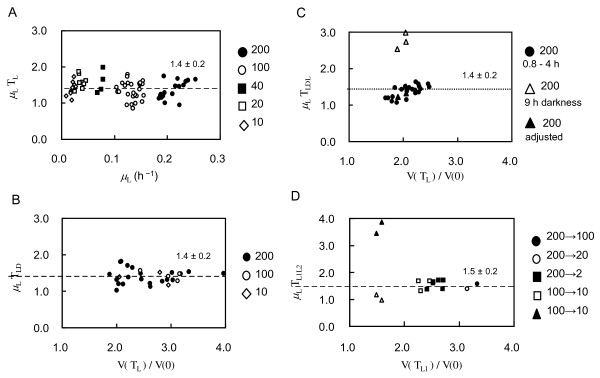
**Product of the rate of cell volume increase and the duration of interdivision phase measured under different illumination conditions**. **A**: LL conditions. Light intensities are as indicated (PAR values are in m^-2 ^s^-1^). **B**: LD conditions. Light intensities are as indicated (PAR values are in m^-2 ^s^-1^). **C**: LDL conditions. PAR was 200 μmol m^-2 ^s^-1 ^in all cases. Various light exposure patterns were used, the duration of the dark periods ranged from 0.8 to 4.0 hours (filled circles), and 9.0 hours (three open triangles). If the value of *T_LD _*is adjusted by subtracting the time cells spent in the dark (*T_D_*), the product of *μ_L_T_LL_*, which is equal to *μ*_*L*(_*T_LDL _*- *T_D_*), becomes approximately equal to 1.4 (three filled triangles) where *T_LL _*is the sum of first and second light period duration. **D**: L1L2 conditions. The intensities of light exposure were lowered (from 200 to 100, 200 to 20, 200 to 2, and 100 to 10 μmol m^-2 ^s^-1^) at various times. For all ratios *V(T_L1_)/V(0) *< 2, the product of *μ_L1_T_L1L2 _*increased far beyond 1.5 (two filled triangles). If we substitute *μ_L1_T_L1L2 _*with *μ_L_T_L1 _*+ *μ_L2_T_L2_*, the value becomes near to 1.5 (two open triangles) where *μ_L2 _*is the rate of the volume increase during the second light period and *T_L2 _*is the duration of the second light period.

When cells were cultivated under the continuous but variable light exposure conditions (LL, Light-Light), with the PAR ranging from 10 to 200 μmol m^-2 ^s^-1^, their volume increased exponentially (see Figure 7A) but the product of the volume increase rate *μ_L _*and the duration of interdivision phase *T_L _*remained constant (*μ_L_T_L _*= 1.4 ± 0.2, mean ± SD, see Figure 8A). Because *μ_L_T_L _*is dependent on the ratio of final cell volume to initial cell volume:

(6) $$\mu_{L}T_{L}=\ln\left(\frac{V(T_{L})}{V(0)}\right),$$

and if *μ_L_T_L _*= 1.4, we conclude that a mother cell is destined to enter the division phase to produce four daughter cells when it had grown to 4.1 times its initial volume. These results suggest that the time at which a *Chlamydomonas *cell enters the division phase is regulated by a 'sizer'.

#### Cell cultivation under discontinuous illumination (LD, Light-Dark) conditions

We then examined whether cells would enter the division phase under discontinuous illumination (LD) conditions, i.e. if the cells are devoid of light before their volume increased to the critical threshold level of 4.1 times their initial volume (Figure 7B). If the time at which the division phase is entered were regulated only by their size, cells would stop growing and their cell cycles would stop progressing in absence of any illumination. If cells could enter the division phase in the absence of illumination and growth, they should have another mechanism to regulate the timing of cell division. Under our experimental settings cells stopped growing when illumination (200 μmol m^-2 ^s^-1^) was switched off, they maintained their volume for some time, but eventually entered the division phase at the time indicated by the white arrowhead in Figure 7B. There *V(T_L_)/V(0) *is the ratio of the cell volume measured at the time of switching the light off to the initial cell volume, *T_L _*is the duration of the light exposure. *V(T_LD_)/V(0) *is the ratio of the final cell volume to the initial cell volume, *T_LD _*is the interdivision phase duration under the LD condition, and *μ_L _*is the rate of cell volume increase during the light exposure.

Cell exposure to light was stopped at various times as shown in Figure 7B. Different PARs were tested for cell cultivation, including 200 μmol m^-2 ^s^-1 ^(N = 27), 100 μmol m^-2 ^s^-1 ^(N = 7) and 10 μmol m^-2 ^s^-1 ^(N = 3). Cells devoid of light always stopped growing but eventually entered the division phase even though they had not grown to 4.1 times their initial volume (*V(T_L_)/V(0) *< 4.1). The product of cell volume growth rate and the duration of interdivision duration *μ_L_T_LD _*was 1.4 ± 0.2, regardless of illumination timing and PAR as long as *V(T_L_)/V(0) *> 2, see Figure 8B. These results suggest that the timing of entering the division phase is controlled not only by a 'sizer' but also by another mechanism that is sensitive to the rate of cell growth (rate of cell volume increase). This mechanism triggers a cell to enter the division phase at an interdivision time *T *= 1.4/*μ*. We call this new interdivision control mechanism the 'interdivision timer'.

### Regulation of the interdivision phase by a volume-based 'interdivision timer'

We also investigated whether re-exposing cells to light would have an effect on the duration of the interdivision phase by the 'interdivision timer'. This was examined using a Light-Dark-Light sequence (LDL conditions). As shown in Figure 7C, re-illuminated cells restarted their growth from the point they had reached before the illumination (200 μmol m^-2 ^s^-1^) stopped, and eventually entered the division phase. Similarly to the earlier introduced ratio *V(T_L_)/V(0)*, where T_L _was the duration of the first light period, we can define *V(T_LDL_)/V(0) *as the ratio of the final cell volume to the initial cell volume, where *T_LDL _*is the duration of the interdivision phase under the LDL conditions. We found that the rates of cell volume increase *μ_L _*during the light periods before and after the dark period were virtually the same. We tested various illumination stop and restart points, with the darkness periods varying between 0.8 and 4.0 hours, see Figure 8C. The product of the volume increase rate *μ_L _*and interdivision phase duration *T_LDL _*was constant at 1.4 ± 0.2. The re-exposure of cells to light and the timing of darkness periods had little effect on the regulation of the duration of interdivision phase by the 'interdivision timer' as long as *V(T_L_)/V(0) *> 2. The initial cell volume was 79.7 ± 12.6 μm^3 ^(mean ± SD).

We also examined the effect of reducing PAR during cell growth at continuous lighting conditions (L1L2) on the duration of the interdivision phase by the 'interdivision timer'. As illustrated in Figure 7D, cell growth slowed when PAR was reduced. Similarly to the earlier introduced ratio V(T_L_)/V(0), where T_L _is the duration of the first light period, we can define *V(T_L1_)/V(0) *as the ratio of cell volume at the end of the L1 period to the initial cell volume, where *T_L1 _*is the duration of the first light period. Similarly, *V(T_L1L2_)/V(0) *is the ratio of final cell volume to initial cell volume, where *T_L1L2 _*is the duration of the interdivision phase (the end of the L1L2 period). A number of different *T_L1 _*and *T_L1L2 _*settings were tested (N = 12), see Figure 8D. We found that the product of the rate of cell volume increase during the first light period, *μ_L1_*, and the duration of the interdivision phase *T_L1L2 _*remains constant at 1.5 ± 0.2. Changes to the PAR had little effect on the 'timer' when *V(T_L1_)/V(0) *> 2.

Overall, the results obtained under several illumination conditions (LL, LD, LDL, and L1L2) indicate that there is an 'interdivision timer' regulating the time at which *Chlamydomonas *cells enter the division phase and that the duration of that interdivision phase *T *is reverse proportional to the rate of cell volume growth and their product remains constant *μT *= 1.4.

### Onset of the next cell cycle during the interdivision phase is regulated by the 'commitment sizer'

Under LD conditions cells did not enter the division phase for 48 hours following the onset of darkness if *V(T_L_)/V(0) *< 2 (Figure 8B). This suggests that there is a threshold cell volume ratio for entering the division phase and that cell can divide only if their volume at the end of the light exposure period was more than twice their initial volume.

Under LDL conditions, when *V(T_L_)/V(0) *< 2 and the duration of dark period was 9 hours, the value of *μ_L_T_LDL _*increased far in excess of 1.4 (see three open triangles in Figure 8C). However, under continuous illumination *μ_L_T_LL _*became nearly 1.4 (see three filled triangles in Figure 8C) where *T_LL _*is the sum of the durations of the first and second light periods. It is evident that a 1.5-h period of darkness introduced before *Chlamydomonas *cells commit to division does not increase the duration of the interdivision phase. This means that the regulation of the onset of division phase by *μT *model will not work if *V(T_L_)/V(0*) < 2.

Under L1L2 conditions, when the cell volume at the time of the change in PAR was less than twice its initial volume (*V(T_L1_)/V(0)*) < 2.1), the value of *μ_L1_T_L1L2 _*increased above 1.5 (two filled triangles in Figure 8D). However, if the value of *μ_L1_T_L1L2 _*is substituted with *μ_L1_T_L1 _*+ *μ_L2_T_L2_*, where *μ_L2 _*is the specific volume growth rate during the L2 period and *T_L2 _*is the duration of the L2 period, this values becomes near to 1.5 (two open triangles on Figure 8D).

Overall, the results obtained under several illumination conditions (LD, LDL, and L1L2) indicate that there is a mechanism that decides whether to commit to the next cell cycle phase by determining if *V(t)/V(0) *> 2. We call this mechanism the 'commitment sizer'.

### Regulation of division number by the 'mitotic sizer'

**Figure 9:**
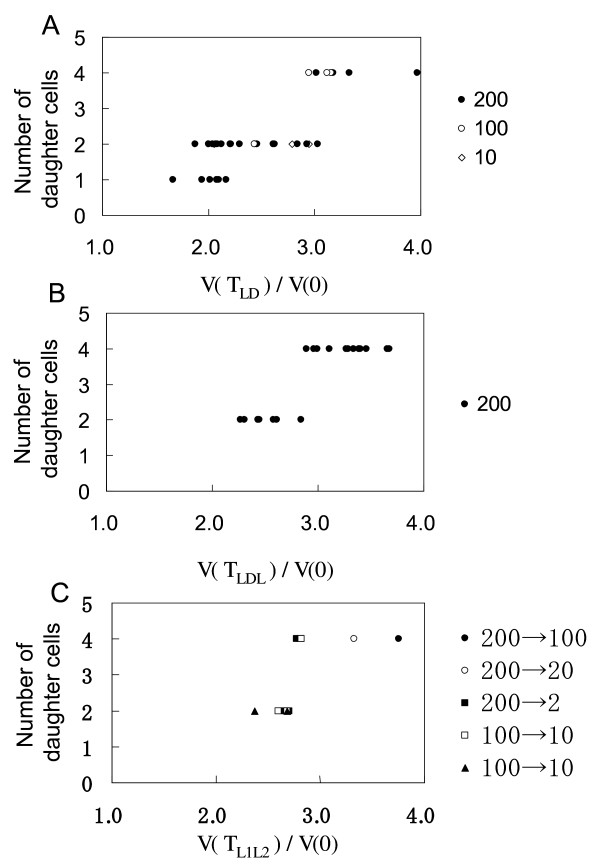
**Effect of light exposure on the number of daughter cells**. **A**: LD conditions. Light exposure was the same as in Fig. 8B. **B**: LDL conditions. PAR and the light exposure patterns were the same as in Fig. 8C. **C**: L1L2 conditions. PAR and the timing of L1 and L2 periods were the same as in Fig. 8 D. Light intensities are as indicated (PAR values are in m^-2 ^s^-1^), all panels.

Under LL conditions mother cells grew up to 4.1 times its initial volume and produced four daughter cells, regardless of the PAR (Figure 5). We set out to investigate how many daughter cells would be produced if the volume of the mother cell has not reached 4.1 times the initial volume by the end of the illumination period. Under LD conditions the number of daughter cells (division number) was determined by the ratio of the final cell volume to the initial cell volume, *V(T_LD_)/V(0) *(Figure 9A). When the value of *V(T_L_)/V(0) *was below 1.8, the mother cell did not enter the division phase even if more than 48 hours passed. If the value of *V(T_LD_)/V(0) *was between 2.2 and 2.8, mother cells divided once and produced two daughter cells. When the value of *V(T_LD_)/V(0) *was above 3.1, mother cells divided twice and produced four daughter cells. Division number varied if the value of *V(T_L_)/V(0) *was between 1.8 and 2.2 (no division or two daughter cells) or between 2.8 and 3.1 (either two or four daughter cells). These results were the same under various PAR conditions (200, 100, and 10 μmol m^-2 ^s^-1^).

Similarly to LD conditions, the ratio of the final cell volume to the initial cell volume, *V(T_LDL_)/V(0)*, determined the number of daughter cells under LDL conditions (Figure 9B). Mother cells divided once and produced two daughter cells if this value was between 2.3 and 2.9; whilst if it was above 2.9, mother cells divided twice and produced four daughter cells. The timing and duration of light exposure and darkness periods had little effect on the 'mitotic sizer'.

Similarly to LD and LDL conditions, the ratio of the final cell volume to the initial cell volume, *V(T_L1L2_)/V(0)*, also determined the number of daughter cells under L1L2 conditions (Figure 9C). If it was below 2.7, mother cells divided once and produced two daughter cells, whilst if above 2.7, mother cells divided twice and produced four daughter cells. PAR and the timing of the L1 and L2 periods had little effect on the 'mitotic sizer'.

Results obtained under several illumination conditions (LL, LD, LDL, and L1L2) thus suggest that there is a mechanism that monitors current cell volume and its increase over its initial value (the initial cell volume), and determines the division number accordingly. For consistency with previously used nomenclature, see e.g. Bisova *et al. *[26], we refer to this mechanism as the 'mitotic sizer'. Previously introduced definition of the 'mitotic sizer', however, is based on the absolute cell volume, whereas ours is based on the relative cell volume (a ratio of the current cell volume to the initial cell volume).

## Discussion

To investigate cell growth and cell cycle progression in the G_1 _phase and their regulation in the eukaryotic cell cycle, we used an on-chip single-cell cultivation system and *Chlamydomonas *cells as the model system. The *Chlamydomonas *cell cycle was divided into interdivision and division phases based on the changes in cell shape (Figure 3). Changes in PAR markedly affected the duration of the interdivision phase but had little effect on the duration of the division phase or its subphases (Figure 4). This is consistent with the results of a previous study in which *Chlamydomonas *and *Chlorella *cells were cultivated conventionally [29]. The time at which the division phase is entered may correspond to the transition point in the *Chlamydomonas *cell cycle [24].

Under the LL conditions cells entered the division phase when they attained 4.1 times their initial volume (Figure 5). When PAR was too small (0.2 μmol m^-2 ^s^-1^), the cells did not grow. This indicates that yeast cells must attain a sufficient growth rate, and in particular a threshold level of protein synthesis [8,30-33]. Our results suggest that a supply of energy in excess of that required for the maintenance of cell metabolism is needed for growth.

The volume of *Chlamydomonas *cells and the growth rate dependence of the cell volume changed in a nonlinear manner (Figure 6A and 6B). Our results are consistent with the results reported for batch cultures of *Chlamydomonas *where the mean cell volume increases exponentially [26,34]. Assuming that the density of cell components remains constant throughout the cell cycle, the cell volume at time *t *in Equation 2 can be replaced by the bulk protein level at time *t*. The bulk protein level in conventionally cultured *Chlamydomonas *cells also increases exponentially [22,35]. The relation between PAR and the cell volume increase (Figure 6C) resembles the relation between PAR and CO_2 _fixation [36,37] and O_2 _production [31]. These results indicate that all these mechanisms (cell growth, CO_2 _fixation, and O_2 _production) are regulated by the rate at which energy is supplied (the ATP production rate) even though the molecular machinery responsible for these mechanisms is located in different parts of the cell. The rate of cell volume increase should be proportional to the total ribosomal content of cells. This has been reported for bacterial cells, in which their ribosome content was proportional to their growth rate when growth was limited by carbon or nitrogen sources during log phase [38], and was also reported for yeast [39-42].

### 'Interdivision timer'

**Figure 10:**
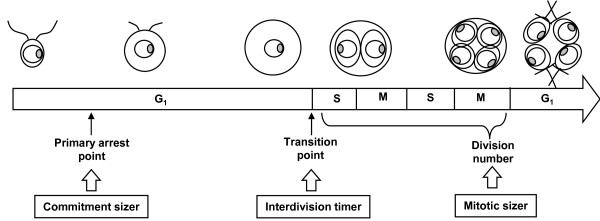
**Cell cycle regulation mechanisms in *Chlamydomonas***. Our results indicate that *Chlamydomonas *cell division progresses through three critical points and that the cell cycle is regulated by 'interdivision timer', 'commitment sizer' and 'mitotic sizer'.

The 'interdivision timer', which depends on the rate at which cell volume increases, determines the duration of the interdivision phase such that *μT *= 1.4 (Figures 8 and 10). Such a timer mechanism was suggested by Donnan and John [22] who used mean protein doubling time in place of the rate at which the cell volume increases. All previous investigators of interdivision timing, however, including Donnan and John, did not consider the cell growth within the cell wall (the 'ready-for-hatch' and the 'growth-after-hatching' subphases, Figure 3), so previously reported results and the definition of the G_1 _phase in *Chlamydomonas *should be reconsidered.

Although we have little information about the 'timer', because cell control mechanisms of this type are rare, we think that it might regulate the G_1_-to-S transition. Using mutants could provide clues to the molecular mechanism behind the 'interdivision timer'. The first such candidate could be the retinoblastoma-related protein deletion strain, *mat3-4 *[24], which should explain the role of the MAT3 protein in this mechanism.

### 'Commitment sizer'

Our results suggest the existence of a 'commitment sizer', which determines whether cells pass through the primary arrest point (Figure 10). Similar situation exists in the cell cycle progression of mammalian cells. Following the mitosis, both *Chlamydomonas and mammalian *cells pass through a control point called the 'restriction point' or 'primary arrest point'. Cells that have already passed the control point stop growing when deprived of energy source (serum or light exposure respectively), but undergo mitosis on schedule. *Chlamydomonas *has also been found to have cell cycle genes similar to those found in the higher plants and animals [26,27].

The effect of photosystem II inhibitor on cell cycle progression might also be taken into account. Light acts mainly in the cell cycle progression of *Chlamydomonas *through photosynthesis and the inhibition of photosynthetic electron transport with DCMU mimics very closely the effects of darkness on the cell cycle [23]. The growth rates of cells cultured under continuous light exposure and then exposed to DCMU was the same as that of cells cultured under continuous light and then subjected to darkness.

Differences between the progression of the mammalian cell cycle and that of the *Chlamydomonas *cell cycle lie in the commitment to division. Thus 1.5 hours of serum depletion immediately before mammalian cells pass through the restriction point usually increases the duration of their interdivision phase by 8 hours [5] whereas devoiding *Chlamydomonas *cell of light did not lengthen the duration of the interdivision phase in green algae.

### 'Mitotic sizer'

It appears that the 'mitotic sizer' (Figure 10) might be monitoring the ratio of final cell volume to the initial cell volume rather than the absolute cell volume. Previous investigators focused on the absolute cell volumes only. Our results are consistent with those of previously published works reporting the correlations between mother cell size and the division number [21] and showing that the division number was determined by the mass of mother cell and was not influenced by the cell growth rate [22]. However, division numbers in previous studies were not even numbers. Asymmetric cell division such that a mother cell produced e.g. three daughter cells never occurred in our experiments. Previously published results reporting odd division numbers could have resulted from incomplete synchronization of the cell cycles in synchronized cultivation experiments, whereas single-cell-based continuous measurement can measure division numbers for individual cells without averaging over the whole of the cell population in batch cultures. We report for the first time that the transition point from two daughter cells to four daughter cells happens at *V(T)/V(0) *ratio being equal to about 3.

As we have mentioned above, the 'mitotic sizer', however, is based on the relative cell volume (current cell volume to initial cell volume), rather than the absolute cell volume. One of the possible mechanisms for recognizing relative volume increase might be a surface-to-volume ratio problem, in other words, the cell growth might reduce the surface tension of cell membrane and might trigger the signalling. However, proving this hypothesis would require monitoring the surface tension of individual cells and its changes during the cell cycle.

In conclusion, we investigated the time-dependency ('timer'), and the size-dependency ('sizer') of *Chlamydomonas *cell division by examining the relation between cell growth and cell cycle progression under different energy supply patterns produced by changing cell exposure to light (duration and PAR). The interdivision time of cells illuminated continuously (LL) was dependent on the cell growth rate and on the reaching of a critical cell size. The product of the rate of cell volume increase and the interdivision time remained constant at 1.4. The interdivision time of cells devoid of light following initial exposure (LD) was also dependent on the cell volume increase rate during illumination and was equal to 1.4 divided by the rate of cell volume increase during the light period. Moreover, the interdivision time of re-illuminated cells (LDL) was similar to that determined for LD cells; re-establishing the light exposure (LDL) had no effect unless the cell size was less than 1.8 times the cell size at the end of the first light exposure. Our results suggest that *Chlamydomonas *cell division has three critical points: 'interdivision timer', 'commitment sizer', and 'mitotic sizer'. During the interdivision phase, the 'commitment sizer' determines whether cells passes through the primary arrest point by monitoring the growth of cells. After cells passed that point, the 'interdivision timer' monitors and memorizes the cell growth rate achieved during the beginning of the light exposure period, it is not affected by the subsequent periods of darkness and restored illumination. During the division phase, the 'mitotic sizer' determines the number of daughter cells according to the ratio of the final cell volume to the initial cell volume.

## Competing interests

The authors declare that they have no competing interests.

## Authors' contributions

KM and AH carried out the experiments, participated in the design of the study and contributed to the drafting of the manuscript. TY and MS participated in the design of the study, the interpretation of results and contributed to the drafting of the manuscript. KY conceived the study, participated in its design and coordination and drafted the manuscript. All authors read and approved the final manuscript.
